# Conceptualizing lifer versus destination patients for optimized care delivery

**DOI:** 10.1186/s12913-023-10214-2

**Published:** 2023-11-01

**Authors:** Jacob Lambrecht, Mahshid Abir, Kristian Seiler, Neil Kamdar, Tim Peterson, Paul Lin, Wilson Nham, Margaret Greenwood-Ericksen

**Affiliations:** 1 Department of Emergency Medicine, University of Michigan, TC B1-220 1500 E Medical Center Dr, Ann Arbor, MI 48109 USA; 2 Acute Care Research Unit, Institute for Healthcare Policy and Innovation, University of Michigan, Ann Arbor, MI USA; 3 RAND Corporation, Santa Monica, CA USA; 4 Data and Methods Hub, Institute for Healthcare Policy and Innovation, University of Michigan, Ann Arbor, MI USA; 5 Department of Emergency Medicine Research, University of Michigan, Ann Arbor, MI USA; 6 Department of Emergency Medicine, University of New Mexico, Albuquerque, NM USA

**Keywords:** Care coordination, Primary care, Specialty care, Healthcare delivery, Healthcare utilization, Ambulatory care, Patient-centered care

## Abstract

**Background:**

Patients presenting to academic medical centers (AMC) typically receive primary care, specialty care, or both. Resources needed for each type of care vary, requiring different levels of care coordination. We propose a novel method to determine whether a patient primarily receives primary or specialty care to allow for optimization of care coordination.

**Objectives:**

We aimed to define the concepts of a Lifer Patient and Destination Patient and analyze the current state of care utilization in those groups to inform opportunities for improving care coordination.

**Methods:**

Using AMC data for a 36-month study period (FY17-19), we evaluated the number of unique patients by residence zip code. Patients with at least one primary care visit and patients without a primary care visit were classified as Lifer and Destination patients, respectively. Cohen’s effect sizes were used to evaluate differences in mean utilization of different care delivery settings.

**Results:**

The AMC saw 35,909 Lifer patients and 744,037 Destination patients during the study period. Most patients were white, non-Hispanic females; however, the average age of a Lifer was seventy-two years whereas that of a Destination patient was thirty-eight. On average, a Lifer had three times more ambulatory care visits than a Destination patient. The proportion of Inpatient encounters is similar between the groups. Mean Inpatient length of stay (LOS) is similar between the groups, but Destination patients have more variance in LOS. The rate of admission from the emergency department (ED) for Destination patients is nearly double Lifers’.

**Conclusion:**

There were differences in ED, ambulatory care, and inpatient utilization between the Lifer and Destination patients. Furthermore, there were incongruities between rate of hospital admissions and LOS between two groups. The Lifer and Destination patient definitions allow for identification of opportunities to tailor care coordination to these unique groups and to allocate resources more efficiently.

## Introduction

Patient-centered care and care coordination have emerged as key areas of focus in healthcare delivery over the last two decades as the United States has focused on improving healthcare quality. Patient-centered care was named by the Institute of Medicine [1] as one of six aims to improve healthcare in the 21st century. Many hospitals and healthcare systems have worked to improve patient outcomes and the continuity of care patients receive. Opportunities for clinical and operations improvement in health systems are often based on the Triple Aim to make care more patient-centered, cost effective, and promote a healthier population [2]. The patient-centered medical home and population health management were devised to optimize care coordination approaches [3, 4].

Healthcare organizations need to understand their patients’ health status, patterns of care utilization, and patient preferences for receiving care in a health system [5–7]. Primary care and specialty care patients have different clinical needs that translate into different requirements regarding the type and intensity of care coordination. This article proposes a novel method of determining the type of care a patient is primarily seen for in a health system—primary versus specialty/destination care. Additionally, there is literature to suggest that having a primary care physician (PCP) reduces all-cause mortality, improves health outcomes such as hospitalizations, and reduces cost of healthcare [8]; therefore, we explore patterns of healthcare utilization between patients with a PCP at the academic medical center (AMC) to patients who receive strictly specialty care to inform investment in care coordination efforts that are optimally responsive to patient needs.

By analyzing patients at the AMC through the Lifer versus Destination lens proposed here, we demonstrate, that the Lifer group will be more consistent visitors to the AMC because they receive primary care services in this setting. In contrast, the Destination group will have more visits to the AMC but have a lower ratio of consistent visitors due to the variable nature of follow-up care required for specialty care patients. We anticipate these findings to be true if tested at similar AMCs or health systems. Further study outside this AMC is needed to confirm the validity of these definitions in other settings.

## Methods

### Setting

A large AMC in the Midwest United States embarked on an examination of primary versus specialty care utilization among its patient population to help guide the health system’s efforts towards improving care coordination and patient-centered care. This AMC includes a tertiary-care hospital, clinics, outpatient surgery centers, and inpatient psychiatric hospital, hence the patient population served is a blend of patients receiving primary care, specialty care, or both.

### Data source

A population-based study was completed by collecting patient encounter, demographics, and residence location data over a 36-month period from FY17-19 from the hospital’s build of the Epic Systems electronic health record (EHR) through the report generator built into the software [9]. All patients that obtained medical care at the AMC during that time were included in the analyses. All protocols were carried out in accordance with all relevant AMC policies and federal regulations.

### Exposure

An analysis examining the number of unique patients by zip code of residence was performed. The patient zip code listed at the most recent encounter was used for the analysis. In the absence of literature defining a Lifer versus Destination patient, we defined these as follows for the purposes of this analysis. A Lifer Patient is a patient who had at least one primary care visit within the study’s window. A primary care visit was defined as an ambulatory care visit with any of the following medical services: internal medicine, family medicine, general pediatrics, adolescent medicine, or the Regional Alliance for Healthy Schools.^1^ This definition is consistent with previous work defining a PCP as a provider in those medical specialties [8, 10]. In contrast, a Destination Patient was defined as a patient with at least one specialty care visit and no primary care service visits in the 36-month window. A patient visit was considered specialty care if the patient saw any ambulatory care service or department not included in the Lifer patient definition.

### Analysis

After the initial analysis of patient origin by zip code, additional demographic information for the Lifer and Destination patient groups, including gender, race, ethnicity, and age were pulled from the EHR. The number of unique patients and health system encounters for Lifer and Destination patients was tabulated. Healthcare utilization data for each group was collected from the EHR to analyze the number of encounters across all healthcare delivery settings including Emergency Department (ED), Inpatient (IP), and Ambulatory Care (AC). AC encounters were further categorized into subgroups by visit type. Utilization data was prepared as number of encounters by setting and as a percentage of all encounters for Lifer and Destination patients.

Data was pulled from the EHR to measure the number of IP encounters for Lifer and Destination patients. Data on 30-day readmissions was subsequently evaluated at the encounter and patient levels, on Lifer and Destination readmissions: (1) the number of index inpatient encounters and the number of 30-day readmissions and (2) the number of Lifer and Destination patients that had IP stays and number of those patients with 30-day readmissions. Inpatient readmission rates were calculated by dividing the number of readmission by the total inpatient encounters. The number of Lifer and Destination patients with readmissions was divided by the total number of inpatient encounters for each group to calculate readmission rate. Mean, standard deviation, and interquartile ranges for IP length of stay (LOS) were also calculated to analyze differences in hospitalization between the groups.

### Statistical analysis

Cohen’s d coefficients were calculated to compare the mean utilization in different healthcare settings (ED, IP, AC) between the average Lifer and Destination patient. Cohen’s d statistics are used when analyses are sufficiently overpowered due to large sample sizes which is common in EHR studies involving large number of encounters and patients. Previously published work provided guidelines around meaningful differences when traditional statistical testing might detect significant differences that are not clinically meaningful [11]. Pooled standard deviations and Bessel’s correction were used to calculate appropriate and weighted Cohen’s d effect sizes. Cohen’s h effect sizes were calculated to measure the difference in proportions between Lifer and Destination patient groups.

Heat maps were created to display geographic distribution by zip code of all patients, Lifer patients, and Destination patients across the state (Fig. 1). The patient density distribution was determined by counting the number of unique patients seen within the health system from a zip code divided by the total population in the zip code. These densities were divided into five quantiles. Quantile 1 (green dots) represents areas of lowest patient density and increase in patient density through quantile 5 (red dots). Grey dots represent zip codes with no patients in that group. The circle represents radial distance from the AMC, which is noted by the star on the maps.

## Results

Over the 36-month study period, 35,909 unique Lifer patients and 744,037 unique Destination patients received care from the AMC. In both groups, majority of the patients were white females of non-Hispanic ethnicity. Of note, there was a sharp age difference between the two groups with the mean age of Lifer patients being 72 years and the mean age of Destination patients being 38 years. Table 1 includes the breakdown of demographic information for these groups.

**Table 1:** Demographic characteristics of patients by Lifer vs. Destination categories

Variable	Lifer Patient		Destination Patient	
	n	%	n	%
Patients	35,909	100%	744,037	100%
Gender				
Female	19,833	55.23%	401,145	53.91%
Male	16,075	44.77%	342,814	46.07%
Unknown	1	0.00%	78	0.01%
Race				
American Indian or Alaska Native	55	0.15%	2,198	0.30%
Asian	1,719	4.79%	34,626	4.65%
Black or African American	2,333	6.50%	68,043	9.15%
Native Hawaiian or Pacific Islander	14	0.04%	561	0.08%
White or Caucasian	30,820	85.83%	581,503	78.16%
Multi-racial	114	0.32%	13,449	1.81%
Other	482	1.34%	23,863	3.21%
Patient refused	101	0.28%	4,003	0.54%
Unknown	271	0.75%	15,791	2.12%
Ethnicity				
Hispanic	449	1.25%	26,423	3.55%
Non-Hispanic	33,213	92.49%	685,954	92.19%
Patient refused	296	0.82%	3,783	0.51%
Unknown	1,926	5.36%	19,268	2.59%
Missing	25	0.07%	8,609	1.16%
Age (years)	Mean (SD)	Median	Mean (SD)	Median
	71.98 (7.19)	70.13	38.21 (24.21)	38.10

Our analysis showed that the health system provides healthcare to a greater number of Destination patients than Lifer patients. Further, Destination patients have a higher number of care encounters than Lifers. Over the study period, the Lifer patients were seen across 672,915 health system encounters; compared to the Destination patients with a total of 4,476,682 encounters. Encounters per patient-year are included in Table 2.

**Table 2:** Patient and health system encounters stratified by lifer versus destination categories (2016–2019)

	Lifer Patients		Destination Patients	
Fiscal Year	Unique Patients	Encounters	Unique Patients	Encounters
2017	31,791	214,367	419,642	1,398,148
2018	32,082	226,496	444,662	1,498,362
2019	31,853	232,052	459,952	1,580,172

FY17 contains records from October 2016 to September 2017, etc

In the analysis of patient origin by zip code, patients came to the health system from across the state, with many patients concentrated in the same region as the AMC. Across all patient groups, most patients resided in the same county as the health system or came from a neighboring county. Lifer patients had a higher proportion of patients residing in or one county away from the health system compared to Destination patients. While many Destination patients reside in or within one county of the health system, the number of Destination patients outside of a two-county radius is higher compared to Lifer patients. This distinction is best seen in Fig. 1 on the patient density heat maps for overall patients, Lifer patients, and Destination patients. These maps reveal that even though the region consistently had the highest patient volumes, there were fewer Lifer patients and a greater number of Destination patients outside of the area.

**Fig. 1a:**
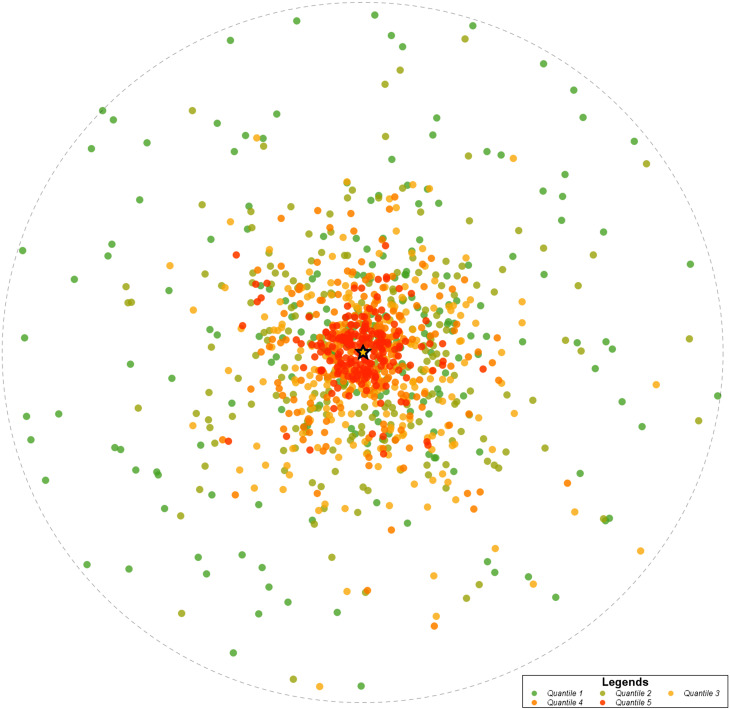
Patient density heat map by zip code of all patients

**Fig. 1b:**
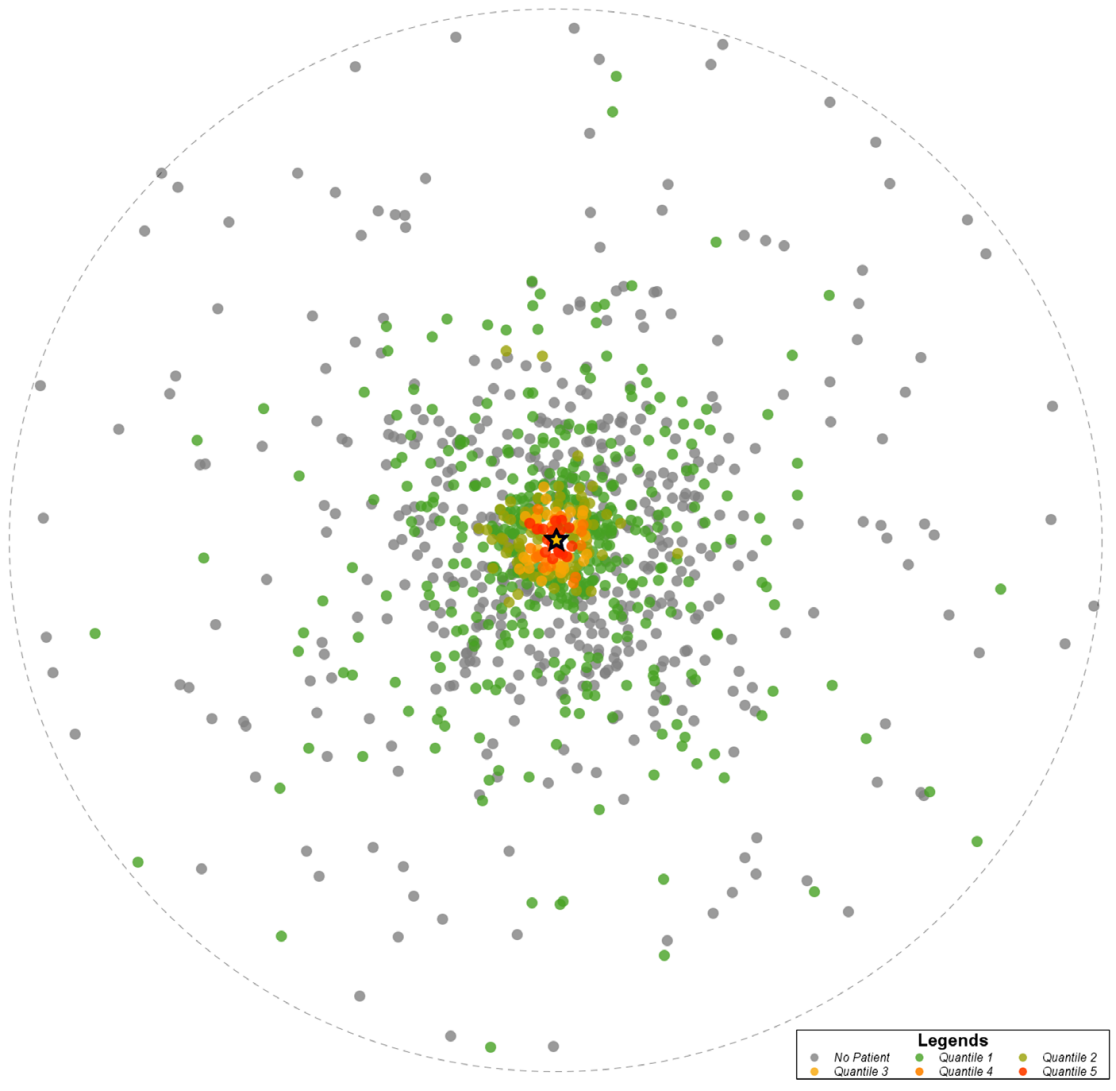
Patient density heat map of Lifer patients by zip code

**Fig. 1c:**
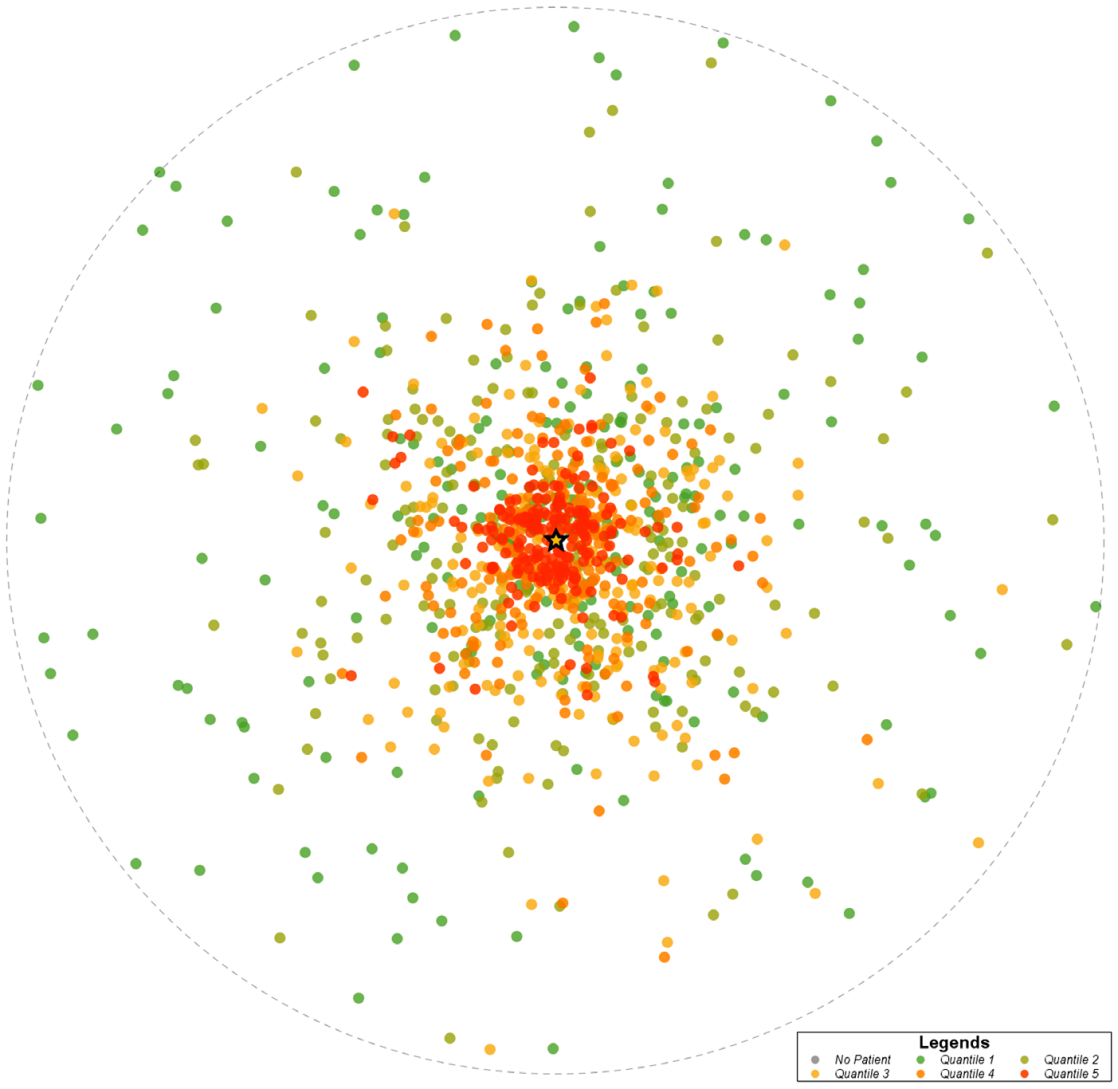
Patient density heat map of Destination patients by zip code

For both patient groups, most patient encounters were AC visits. A Lifer patient had an average of 17.65 AC visits compared to an average 5.36 AC visits for a Destination patient. The difference in means for AC visits is large, with Cohen’s effect size *d* of 1.31 Within the AC encounter sub-groups, there was a small difference (Cohen’s *d* of 0.22) in utilization of virtual care between the two groups. Lifer patients had an average of 0.29 virtual visits over the study and all Lifers contributed a total of 10,578 virtual visits. In comparison, a Destination patient had an average of 0.03 virtual care visits in the same period and all Destination patients had a combined 24,591 virtual visits.

Destination patients have a higher proportion of ED encounters at 6.62%, whereas ED encounters comprise 3.81% of all Lifer encounters. The effect sizes for ED encounters demonstrate a small difference (Cohen’s d of 0.38) between the two groups and the proportion of ED visits is lower in the Lifer group (Cohen’s h of -0.13). Furthermore, the proportion of ED visits that resulted in hospital admission were higher in the Destination cohort at 4.72% in contrast to 1.98% for Lifers. The overall rate of inpatient hospitalization (IP) between Lifer and Destination patients was relatively similar, with IP stays accounting for 3.05% versus 4.23% of all encounters for each group, respectively. A Lifer patient had an average of 0.57 IP visits during the study period whereas a Destination patient had an average of 0.25 IP visits. Cohen’s effect size reveals a small difference (d = 0.34) between the means. Table 3 contains the complete breakdown of healthcare utilization by setting between the patient groups and can be found at the end of the document.

**Table 3:** Utilization by care delivery setting for Lifer and Destination patients

Variable	Lifer Patients				Destination Patients					
Value	n	%	Pt mean	Std dev	n	%	Pt mean	Std dev	Cohen’s d^3^	Cohen’s h^4^
Encounters	672,915	100			4,476,682	100				
Setting										
AC	633,624	94.16%	17.65	15.49	3,989,496	89.12%	5.36	8.01	1.31**	0.18
IP	20,541	3.05%	0.57	1.36	189,547	4.23%	0.25	0.86	0.34*	-0.06
Length of Stay^1^			4.45	5.57			5.06	9.08	0.08	
Readmission^2^	3,710	18.06%	0.37	1.03	29,082	15.34%	0.24	0.97		0.07
ED	25,669	3.81%	0.37	1.14	296,577	6.62%	0.11	0.61	0.38*	-0.13
ED to admit	13,335	1.98%	0.34	1.03	211,161	4.72%	0.28	0.96	0.06	-0.16
ED to discharge	12,334	1.83%	0.71	1.78	85,416	1.91%	0.40	1.27	0.23*	-0.01
Psych	535	0.08%	0.01	0.44	26,757	0.60%	0.04	0.88	0.02	-0.10
Obs	3,274	0.49%	0.09	0.38	24,054	0.54%	0.03	0.24	0.23*	-0.01
OP Surgery	6,416	0.95%	0.18	0.83	86,478	1.93%	0.12	0.97	0.06	-0.08
OP in a bed	2,838	0.42%	0.08	0.31	25,375	0.57%	0.03	0.21	0.20*	-0.02
AC Encounter Type										
Clinical Support	21,957	3.26%	0.61	2.13	107,488	2.40%	0.14	1.01	0.37	0.05
Office	590,809	87.80%	16.45	13.98	3,802,530	84.94%	5.11	7.57	1.30**	0.08
Patient Outreach	7,886	1.17%	0.22	1.53	8,137	0.18%	0.01	0.28	0.19	0.13
Pre/Postnatal	1	0.00%	0.00	0.01	30,443	0.68%	0.04	0.34	0.12	-0.16
Initial Prenatal	1	0.00%	0.00	0.01	9,862	0.22%	0.01	0.13	0.11	-0.09
Routine Prenatal	0		0.00	0.00	7,931	0.18%	0.01	0.16		-0.08
Postpartum	0		0.00	0.00	12,650	0.28%	0.02	0.15		-0.11
Virtual	10,578	1.57%	0.29	1.96	24,591	0.55%	0.03	0.53	0.22*	0.10
Telephone	10,462	1.55%	0.29	1.96	22,156	0.49%	0.03	0.52	0.22*	0.11
Video	116	0.02%	0.00	0.06	2,435	0.05%	0.00	0.07	0.00	-0.02
Procedure	2,393	0.36%	0.07	0.37	16,307	0.36%	0.02	0.19	0.20*	0.00

^1^Encounter level (instead of patient) means and standard deviations are reported

^2^Percent shown as percent of inpatient cases, patient means calculated using patients with at least one inpatient encounter

^3^ * represents small effect size, ** represents a large effect size

^4^Directionality has been preserved (negative values indicate proportion is lower in Lifer group)

The average IP LOS is 4.45 and 5.06 days for Lifer and Destination groups, respectively. While the difference between the mean LOS is negligible (Cohen’s d of 0.08), the standard deviations vary, at 5.57 days for Lifer and 9.08 days for Destination groups. Interquartile range and other dispersion statistics for LOS are presented in Table 4.

**Table 4:** Dispersion of inpatient length of stay (in days) between groups

	N	Min	p1	p5	Q1	median	Q3	p95	p99	Max
Lifer	20,541	0.01	0.49	0.88	1.30	2.83	5.29	13.80	27.76	115.23
Destination	189,547	0.00	0.42	0.88	1.36	2.63	5.25	16.72	38.78	512.50

The Lifer group had 18.06% case-level and 20.74% patient-level 30-day readmission rates. In comparison, the Destination group had 15.34% case-level and 13.35% patient-level 30-day readmission rates. Lifer patients had higher rate of 30-day readmissions at both levels. Table 5 displays the complete breakdown of 30-day readmissions.

**Table 5:** 30-day readmission rates by Lifer Status

	Encounter Readmission Rate			Patient Readmission Rate			
Lifer	Inpatient Encounters	Readmissions	Readmission Rate	Total Patients	Inpatient Encounters	Readmissions	Readmission Rate
0	189,547	29,082	15.34%	744,037	120,924	16,141	13.35%
1	20,541	3,710	18.06%	35,909	10,094	2,093	20.74%

## Discussion

All patients seen at this health system primarily reside in southeastern part of the state, regardless of which patient group they belong to. However, there is a decrease in the number of Lifer patients outside of that region and an inversely increasing number of Destination patients from outside of the same region. The most probable explanation of this finding is that Lifer patients live or work near the AMC. Previous work examined patients’ preferences in receiving primary care using a five-point scale and recorded percent of responses that rated that characteristic of care a 4 or 5. Travel cost was rated 68.3% and geographic proximity to the PCP office was rated 78% [12]. Qualitative work reflected similar themes in patients favoring a PCP office close to their homes [13]. In contrast, this health system is one of only a few AMCs in the state with a range of specialty care services. Consequently, the AMC sees patients from across the state for specialty care but fewer patients from outside of the one-county radius for primary care.

The health system’s patients are predominantly Caucasian, females, of non-Hispanic ethnicity. These data closely mirror demographic data for the region based on the 2010 U.S. Census [14]. We noted a stark difference in age between Lifer and Destination patients. The mean age of a Lifer patient was about seventy-two years while the mean age of a Destination patient was thirty-eight years. This finding was not anticipated but is somewhat supported by current literature. Younger adults are less likely to have a PCP and only seek care when they have a medical concern. This has been attributed to several factors including perceived low risk for disease, financial instability or lack of insurance while transitioning between life stages, and poor transitions from pediatric to adult medical providers [15, 16].

If a patient does not have any chronic medical conditions and has completed their vaccinations, they will have fewer reasons to see a PCP. As a patient transitions to an adult provider, they are also less likely to have adequate insurance coverage. For instance, coverage under the Children’s Health Insurance Program [CHIP] typically stops at 19, but children with disabilities or complex medical needs may be eligible for Medicaid longer [17]. For patients with complex, chronic health problems, the opposite would likely be true. Sick, young adults will need more access to care and may have extended insurance coverage. While this population may still see a PCP, they may also see specialists to manage these conditions. Because younger patients may not have any primary care visits, this group may skew towards the Destination cohort. This age group may be at higher risk to present to emergency care for flares of chronic medical conditions. An example of this was demonstrated in a recent study, which found that adolescents with sickle cell disease, a chronic disease requiring complex care management, had higher healthcare utilization, specifically in the ED and IP settings [18]. Patients with multiple medical diagnoses were shown to have increased use of healthcare services [19]. Thus, care coordination for Destination patients may need to focus on reducing need for emergency care and hospitalizations and ensuring availability of rapid access and follow-up with appropriate healthcare providers, but more evidence is needed in this regard.

The Lifer category included 35,909 unique patients in the study period. As illustrated by Table 2, Lifer’s annual unique patient count for each study year is very close to the total unique patient count. In contrast, there were 744,037 unique Destination patients during the study. Annual unique patient counts for the Destination group averaged just over 50% of the total patient count. This finding reinforces the notion that Lifer patients, as defined in this study, are the most consistent visitors to the health system.

Most of the health system’s patient encounters occurred in the AC setting for both Lifers and Destination patients. Destination patients had a greater number of visits to AC offices; however, Lifer patients had a higher number of average visits. This could be because Lifer patients see primary care services at the AMC in addition to medical and surgical specialties. Data for virtual care appears to demonstrate Lifer patients having more virtual care visits. However, Cohen’s effect size for the means is small, so Destination use of virtual care may just appear lower simply because there are more Destination patients. Ultimately, virtual care utilization appears to be low in both groups; therefore, the current capacity of virtual care should be examined and the possibility of increasing virtual care should be considered. Investigations into patients’ choices and acceptance of virtual care as a modality of healthcare delivery has already begun in primary and specialty care settings [20–23]. We suggest prioritizing expansion of virtual care services to both groups as indicated by patient preference at this AMC.

The difference in ED utilization by Lifer versus Destination patients was small but higher among Destination patients. It is worth investigating the higher rate of ED encounters that result in admission amongst the Destination cohort. Admission rates for Destination patients could be higher because the state of care coordination and timely follow-up care may be unknown and consequently considered less robust. Therefore, hospitalization or observation could be considered a safer choice than outpatient follow-up care.

Inpatient hospitalization rates are comparable for the two groups. The difference in the mean number of IP visits was trivial between the Lifer and Destination groups. Hospital readmission rates are measured by the Centers for Medicare and Medicaid Services [CMS] as a quality indicator for care coordination. CMS value-based purchasing programs indicated a hospital readmission within 30 days of discharge from an IP setting, regardless which hospital the patient is readmitted to, is a prognosticator of suboptimal care coordination [24]. Lifer patients had higher 30-day readmission rates than Destination patients. While the exact cause of this result is not clear, it is probably at least partially due to the fact that we are using internal data and cannot know the true readmission rate for Destination patients that could include admission to hospitals unaffiliated with the AMC. The same logic could be extended to ED utilization for Destination patients. It is unlikely however, that AC visits are impacted in a similar fashion because the specialty care follow-up appointment would be at the AMC. Lifer patients having a higher readmission rate warrants further investigation by the AMC. This would allow for recognition of gaps in care coordination, thus lowering the readmission rate for all patients.

Mean hospital LOS, measured in days, appears similar between the two groups and the effect size is small. Dispersion data demonstrates LOS is similar amongst the groups until the 95th percentile, at which point there appears to be greater variation in LOS among Destination patients. While this suggests the differences in LOS are mostly outliers, there is still potential significance to the longer LOS in this group as prolonged hospitalization increases the risk of developing hospital-acquired infections, pressure injuries, and other complications. It is unclear whether the skewed variance in LOS can be attributed to Destination patients undergoing more complex procedures such as organ transplant, thus requiring longer monitoring or if Destination patients are sicker on transfer, thereby requiring longer hospitalizations. Further investigation by the AMC may be warranted.

These findings can inform decisions regarding care coordination for both groups. A Lifer patient has more AC visits but a lower ED utilization and IP admission rate. In contrast, a Destination patient has fewer AC visits, higher ED utilization, higher IP admission, and potentially longer IP LOS. This may suggest that Lifer patients have more robust care coordination and/or require less intensive resources to manage their care. Care of the Destination patient uses more resources and a higher level of care. For Destination patients, improving care coordination to reduce ED visits and IP hospitalizations is paramount as these encounters are more costly to the patient and could increase resource strain on the AMC [25, 26].

One limitation of this work is that obstetrics and gynecology (OB/Gyn) patients were considered a specialty care service by definition; therefore, making them Destination patients. There was only one Lifer encounter with a pre- or post-natal care visit, which is a sharp contrast to the 30,443 Destination OB encounters. We recognize that at least some patients receiving OB care at the health system are also likely receiving routine primary care. Grouping OB encounters in the Destination patient group could also skew the mean age of Lifer patients upward, as most OB patients are generally anywhere in age from early 20s to late 30s [27].

Another potential limitation is the lack of insurance data available for analysis. While the patient’s type of insurance is less likely to impact access to care in the ED or inpatient setting due to EMTALA, there may be disparities in access to primary or specialty care clinicians for uninsured patients [28, 29]. Patients who are unable to access the specialty care they require in an outpatient setting may present to the ED to receive treatment for their health needs. Therefore, future comparisons between these two groups should explore insurance status as a variable impacting healthcare utilization.

One consideration for this work is the impact of the COVID-19 pandemic on healthcare delivery, specifically the utilization of virtual care pre- and intra-pandemic. COVID-19 affected the ability to provide in-person healthcare for many health systems across the globe. All data discussed herein is reflective of the health system in a pre-pandemic state. The reduction of face-to-face care experienced during the pandemic meant a shift to virtual visits for most appointments. With the pandemic winding down, there has been a return to office visits, which has created a hybrid state of visit types. It would be worthwhile to examine if Lifer and Destination patients’ care delivery preferences at the AMC have changed in response to an increased virtual care capacity.

## Conclusion

The novel Lifer and Destination definitions presented in this work identify two unique patient groups, each requiring different resources within the same AMC. For this AMC, Lifer patients have a higher number of AC encounters than Destination patients. Rate of admission from the ED is higher for Destination patients. Both groups have a comparable proportion of IP encounters, but Destination patients often have longer hospital stays. These definitions can be used to inform care coordination resource allocation among these two groups optimizing care delivery according to their preferences, healthcare needs, and care utilization patterns. While this work shows promise in identifying and optimizing patient-centered care for these patient populations, further research surrounding these two groups is warranted. Future study could focus on analyzing utilization patterns in a post-pandemic era, addressing the limitations of this work, and assessing the broader applicability of these definitions at other health systems and AMCs.

## Data Availability

All data analyzed during this study are summarized in this published article. The patient-level data is not open-access, nor is it associated with a permanent identifier.

## Footnotes

1 The Regional Alliance for Healthy Schools is an initiative sponsored by the AMC that provides a range of healthcare services, including but not limited to primary care, nutrition counseling, dental, mental health care, and tangibles to school-aged children 21 and under in select counties.

## List of Abbreviations

AMC: Academic medical center
AC: Ambulatory care
CDC: Centers for Disease Control and Prevention
CHIP: Children’s Health Insurance Program
CMS: Centers for Medicare and Medicaid Services
ED: Emergency department
EHR: Electronic health record
FY: Fiscal year
IP: Inpatient
LOS: Length of stay
OB/Gyn: Obstetrics and gynecology
PCP: Primary care provider
SD: Standard deviation

## References

[1] Institute of Medicine [IOM] (2001). Crossing the Quality Chasm: a New Health System for the 21st Century.

[2] Berwick DN (2008). The Triple Aim: Care, Health, and cost. Health Aff.

[3] Hollingsworth JM, Saint S, Sakshaug JW, Hayward RA, Zhang L, Miller DC (2012). Physician practices and Readiness for Medical Home reforms: policy, pitfalls, and possibilities. Health Serv Res.

[4] Share DC (2011). How a Regional Collaborative of hospitals and Physicians in Michigan Cut costs and improved the quality of Care. Health Aff.

[5] Johnston R. C. V. (2010). What is known about the effects of medical tourism in destination and departure countries? *Int J Equity Health, 9*(24). 10.1186/1475-9276-9-24 PMC2987953 21047433

[6] Andersen R (1986). Health Status and Health Care utilization. Health Aff.

[7] New England Journal of Medicine [NEJM]. (2017, January 1). What Is Patient-Centered Care? *NEJM Catalyst*.

[8] Starfield B, Shi L, Macinko J. (2005, October 3). Contribution of Primarcy Care to Health Systems and Health. *The Milbank Quarterly, 83*(3), pp. 457–502. 10.1111/j.1468-0009.2005.00409.x PMC2690145 16202000

[9] Epic Systems Corporation (2020). Epic Electronic Health Record.

[10] DuGoff EH, Walden E, Ronk K, Palta M, Smith M (2018). March). Can Claims algorithms identify the physician of record?. Med Care.

[11] Cohen J. Statistical Power Analysis for the behavioral sciences. Academic Press; 2013.

[12] Kenny P, De Abreu Lourenco R, Wong CY, Haas M, Goodall S (2016). Community preferences in general practice: important factors for choosing a general practitioner. Health Expect.

[13] Bazyar M, Yazdi-Feyzabadi V, Bahmani M, Sadeghifar J, Momeni K, Shaabani Z. (2022, September 13). Preferences of people in choosing a family physician in rural areas: a qualitative inquiry from Iran. *Primary Health Care Research & Development, 23*(e57), 1–10. 10.1017/S1463423622000317 PMC9472239 36097717

[14] United States Census Bureau. (2019). *Quick Facts: Michigan, Washtenaw County, Macomb County, Wayne County, Oakland County, Michigan* Retrieved October 2020, from U.S. Census Bureau Quick Facts: https://www.census.gov/quickfacts/fact/table/macombcountymichigan,oaklandcountymichigan,waynecountymichigan,washtenawcountymichigan,MI/PST045219.

[15] Callahan ST (2010). May). Changes in Ambulatory Health Care Use during the transition to Young Adulthood. J Adolesc Health.

[16] Graves L. L. S. (2019, September 1). Transitions of Care for Healthy Young Adults: Promoting Primary Care and Preventative Health. *Southern Medical Journal, 112*(9), 497–499. 10.14423/SMJ.0000000000001017 31485590

[17] Centers for Medicare & Medicaid Services. (n.d.). CHIP Eligibility. Retrieved. July 2023, from CHIP | Medicaid.gov: https://www.medicaid.gov/chip/eligibility/index.html.

[18] Moody KL. (2022, April 4). Healthcare utilization and the quality of life of children and adolescents with sickle cell disease. *Pediatric Blood & Cancer, 69*(8), e29685. 10.1002/pbc.29685 35373909

[19] Frølich A, Ghith N, Schiøtz M, Jacobsen R, Stockmar A. (2019, August 1). Multimorbidity, healthcare utilization and socioeconomic status: A register-based study in Denmark. *PLOS One*, 10.1371/journal.pone.0214183. 10.1371/journal.pone.0214183 PMC6675513 31369580

[20] Sullivan MW, Kanbergs AN, Burdette ER, Silberman J, Dolisca S, Scarry J, Bernstein SN. (2021, September 23). Acceptability of virtual prenatal care: thinking beyond the pandemic. *The journal of maternal-fetal & neonatal medicine, 35*(25), 8472–8475. 10.1080/14767058.2021.1980534 34554895

[21] Stamenova V, Agarwal P, Kelley L, Fujioka J, Nguyen M, Phung M, Bhattacharyya O. (2020, July 6). Uptake and patient and provider communication modality preferences of virtual visits in primary care: a retrospective cohort study in Canada. *BMJ Open, 10*(7), e37064. 10.1136/bmjopen-2020-037064 PMC7342856 32636284

[22] Yedulla N, Faraj MT, Koolmess DS, Battista EB, Montgomery ZA, Day CS (2021). July). Assessing Orthopedic Patient preferences for mandated virtual care during the COVID-19 pandemic and elective virtual care in non-pandemic circumstances. Orthopedics.

[23] Beamish P, McNeill K, Arnaout A, Malcolm J. Patient perspectives on virtual care for Diabetes Management in the era of COVID-19. Can J Diabetes. 2023, July. 10.1016/j.jcjd.2023.07.001. 10.1016/j.jcjd.2023.07.001 37437840

[24] Centers for Medicare and Medicaid Services. (2021, May 8). *Hospital Readmissions Reduction Program (HRRP)*. Retrieved from CMS.gov: https://www.cms.gov/Medicare/Quality-Initiatives-Patient-Assessment-Instruments/Value-Based-Programs/HRRP/Hospital-Readmission-Reduction-Program.

[25] Moore B, Liang L. (. (2020, December). *Costs of Emergency Department Visits in the United States, 2017* Retrieved from HCUP Statistical Brief #268. Agency for Healthcare Research and Quality.: https://hcup-us.ahrq.gov/reports/statbriefs/sb268-ED-Costs-2017.pdf. 33439600

[25] Machlin S, Mitchell E. (2018, October). *Expenses for Office-Based Physician Visits by Specialty and Insurance Type, 2016* Retrieved from Statistical Brief #517. Agency for Healthcare Research and Quality.: https://meps.ahrq.gov/mepsweb/data_files/publications/st517/stat517.shtml. 30395427

[27] Centers for Disease Control and Prevention. (2017, July 7). *Key Statistics from the National Survey of Family Growth - B Listing* Retrieved July 2023, from National Survey of Family Growth - CDC: https://www.cdc.gov/nchs/nsfg/key_statistics/b.htm#agefb.

[28] Creedon TB, Zuvekas SH, Hill SC, Ali MM, McClellan C, Dey JG (2022). Effects of Medicaid expansion on insurance coverage and health services use among adults with disabilities newly eligible for Medicaid. Health Serv Res.

[29] Skinner AC, Mayer ML. (2007, November 28). Effects of insurance status on children’s access to specialty care: a systematic review of the literature. *BMC Health Services Research, 7*(1), 194. 10.1186/1472-6963-7-194 PMC2222624 18045482

